# Effects of high-frequency repetitive transcranial magnetic stimulation combined with acupuncture on central pain and mood

**DOI:** 10.3389/fpsyt.2026.1752555

**Published:** 2026-04-27

**Authors:** Xiangyue Wu, Binqing Li, Ting Xiang, Xiaolu Fan

**Affiliations:** 1 Department of Rehabilitation Medicine, The Third People’s Hospital of Hubei Province, Wuhan, Hubei, China; 2 Department of Gastrointestinal Surgery, Hubei Provincial Third People’s Hospital (Zhongshan Hospital), Wuhan, China

**Keywords:** acupuncture, central post-stroke pain, emotion, neuropathic pain, repetitive transcranial magnetic stimulation

## Abstract

**Objective:**

This study aimed to observe the clinical efficacy of multi-target high-frequency repetitive transcranial magnetic stimulation (rTMS) combined with acupuncture for chronic central post-stroke pain (CPSP) and emotional comorbidities during the recovery phase.

**Methods:**

Thirty patients with CPSP admitted to the Rehabilitation Department of Hubei Third People’s Hospital from June 2024 to June 2025 were randomly assigned to an acupuncture group (n=15) and a combined treatment group (n=15). The acupuncture group received routine rehabilitation plus acupuncture; the combined group received, in addition, multi-target rTMS targeting the contralateral primary motor cortex (M1) and the left dorsolateral prefrontal cortex (DLPFC), both at 10 Hz, for 4 weeks. Outcomes were assessed at baseline and after 4 weeks using the Visual Analogue Scale (VAS) for pain, Hamilton Anxiety Scale (HAMA) and Hamilton Depression Scale (HAMD) for mood, Pittsburgh Sleep Quality Index (PSQI) for sleep, and Activities of Daily Living (ADL) for functional status.

**Results:**

After 4 weeks, a linear mixed model (LMM) analysis revealed a significant main effect of Time for all outcomes (VAS, HAMA, HAMD, PSQI, ADL; all P < 0.001), indicating overall improvement across both groups. A significant Group × Time interaction was found for VAS (P = 0.018) and HAMD (P = 0.037), demonstrating that the combined treatment group experienced significantly greater improvements in pain and depression compared to the acupuncture-only group. No significant interactions were observed for HAMA (P = 0.226), PSQI (P = 0.343), or ADL (P = 0.210).

**Conclusion:**

Acupuncture confers clinical benefit in CPSP, whereas the addition of multi-target high-frequency rTMS yields superior analgesic and psychotropic effects. This combined approach has considerable clinical value for patients in the post-stroke recovery phase.

## Introduction

Central post-stroke pain (CPSP) is a neuropathic pain developing typically months after stroke with an incidence of 1–12%, characterized by unilateral stabbing, burning, electric shock-like pain, or numbness and paresthesia. Typical symptoms of CPSP include persistent headache, shoulder discomfort and musculoskeletal pain (1, 2). More seriously, CPSP frequently results in anxiety, depression, and insomnia (3). The chronic pain severely affects patients’ psychological state, rehabilitation progress, and quality of life (4). Currently, CPSP treatment is based on a combination of drug therapy and neuromodulation technology (motor cortex stimulation, deep brain stimulation, spinal cord stimulation), or other treatments, like acupuncture and mirror therapy (5, 6). Traditional Chinese Medicine theory posits that the therapeutic effects of acupuncture and moxibustion stem from stimulating specific points (acupoints) along the meridians (the pathways through which “Qi,” or vital energy, flows to regulate the body’s physiological functions (7), this process relies on reflex arc-mediated activation of peripheral nerves, enabling sensory signals to ascend through the spinal cord to the brainstem and higher centers, thereby modulating the peripheral autonomic nervous system via descending pathways (8). Mechanistically, lectroacupuncture may exert analgesic effects by inhibiting neuronal apoptosis and abnormal astrocyte activation in the brain (9). In addition, subsequent investigations have further revealed acupuncture can alleviate CPSP through modulating neuroinflammatory responses, balancing excitatory and inhibitory neurotransmission, and improving cerebral perfusion (10). This is consistent with Zhang T’s findings on acupuncture for post-stroke thalamic pain (3). Researchers included a total of 12 clinical studies comprising 953 confirmed subjects with post-stroke thalamic pain. The included trials featured sample sizes ranging from 26 to 200 cases and treatment durations of 14–60 days, involving multidimensional interventions including Western medicine and acupuncture. Systematic analysis revealed that acupuncture was more effective than conventional pharmacotherapy in reducing Visual Analog Scale (VAS) scores, pain intensity ratings, and pain rating indices. Repetitive transcranial magnetic stimulation (rTMS) is a non-invasive brain stimulation (NIBS) techniques which applies rapidly changing magnetic fields to alter neuronal membrane potentials and modulate cortical networks. It has been widely utilized in neurological and psychiatric diseases, such as Alzheimer’s disease (11), depression (12) and sleep disturbances (13), and has demonstrated significant efficacy in the treatment of neuropathic pain (5). International clinical guidelines recommend that high-frequency rTMS (HF-rTMS) targeting the primary motor cortex(M1) contralateral to the pain site be used to alleviate neuropathic pain (6). Its analgesic mechanism is closely associated with correcting abnormal cortical excitability, activating descending inhibitory control pathways, and mobilizing the endogenous opioid system. Additionally, the dorsolateral prefrontal cortex (DLPFC), anterior cingulate cortex (ACC), and secondary somatosensory cortex (S2) are potential therapeutic targets (6). Confirming earlier observations, ten sessions of low-frequency DLPFC rTMS significantly alleviate phantom limb pain, with clinical benefits persisting at 2 months (14). Although rTMS stimulation parameters remain inconsistent across studies (15, 16), its target-specific and symptom-specific characteristics have been well established in neuropathic pain treatment. M1 stimulation demonstrates definite advantages in pain relief, effectively reducing neuropathic pain intensity and producing favorable long-term analgesic effects; however, its improvement on emotional symptoms is limited (17, 18). In contrast, left DLPFC stimulation shows no significant short-term analgesic effects, but exerts remarkable medium- and long-term analgesic effects on chronic pain while effectively alleviating accompanying depressive symptoms (19, 20). Given that neuropathic pain commonly presents as multidimensional symptoms including pain, emotional disorders, and sleep disturbances, and single-target stimulation fails to address all these symptom clusters simultaneously, we hypothesize that combined M1 and DLPFC stimulation may concurrently activate sensorimotor and emotion regulation networks, achieving more comprehensive and sustained synergistic intervention for neuropathic pain and its accompanying symptoms. Therefore, this study proposes a combined stimulation protocol as a novel attempt at multi-target rTMS therapy. Furthermore, acupuncture combined with rTMS for NP has gradually gained attention, yet relevant research remains limited. A retrospective study on postherpetic neuralgia patients (21) demonstrated that rTMS plus acupuncture was significantly superior to rTMS alone and nerve block control in pain relief, which may be achieved by inhibiting NOD-like receptor 3 (NLRP3) inflammasome activation and reducing inflammatory factors including tumor necrosis factor-α (TNF-α), interleukin (IL)-1β, and IL-6. However, such integrated Chinese and Western medicine approaches are currently understudied. Building upon previous research, this study attempts for the first time to combine acupuncture with multi-target rTMS stimulation. We hypothesize that this integrated intervention will outperform single-modality treatments, exploring a novel multi-target, multi-mechanism approach for CPSP management and providing more comprehensive therapeutic strategies for clinical practice.

## Materials and methods

### Participants

**Figure 1:**
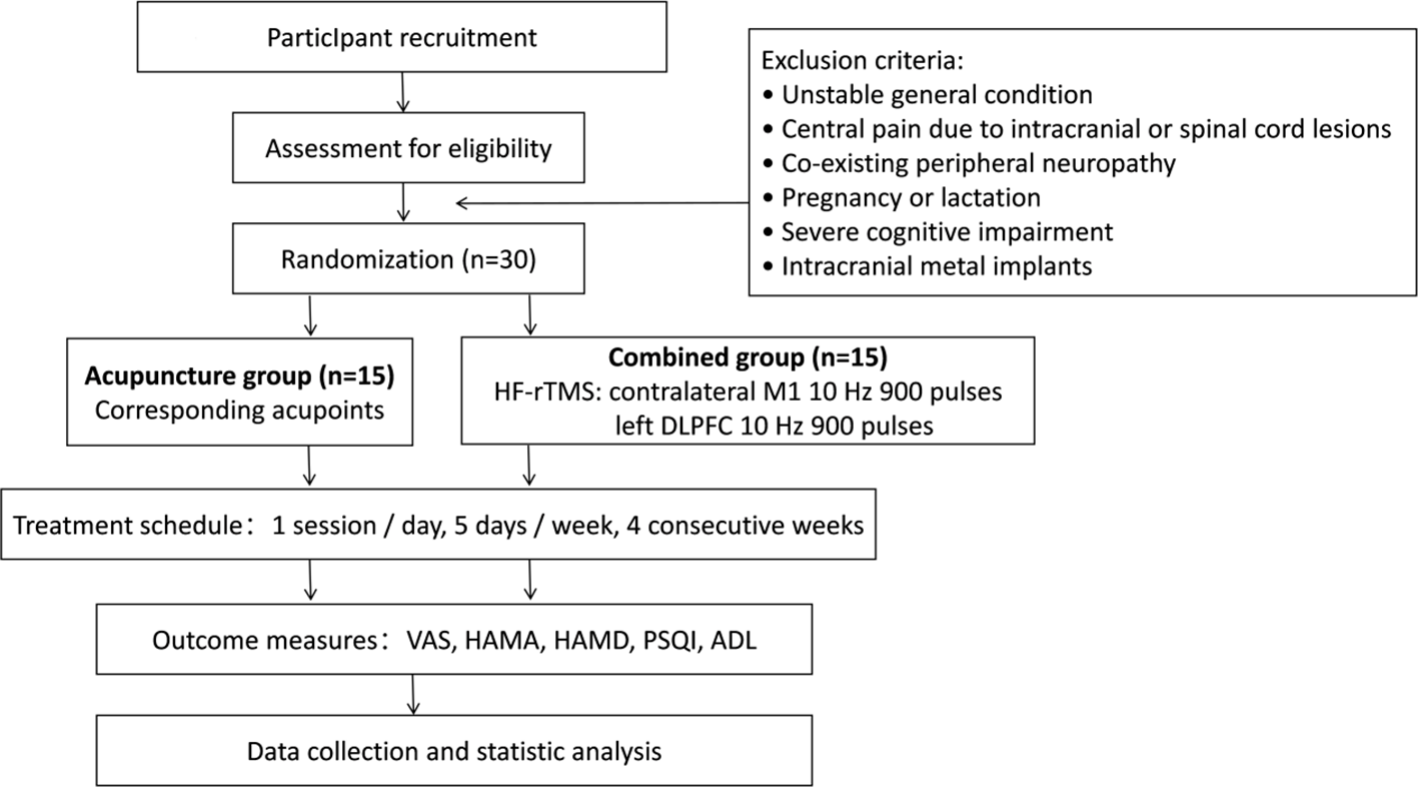
Study design flow chart. ADL, Activities of Daily Living; HAMA, Hamilton Anxiety Rating Scale; HAMD, Hamilton Depression Rating Scale; HF-rTMS, high-frequency repetitive transcranial magnetic stimulation; VAS, Visual Analogue Scale.

Thirty CPSP patients treated in the Department of Rehabilitation Medicine of Hubei Third People’s Hospital between June 2024 and June 2025 were enrolled. Study inclusion criteria: participants must have a confirmed first-time stroke, whether ischemic or hemorrhagic, as verified by neuroimaging (22). Meanwhile, participants are diagnosed with CPSP, and have stable vital signs and clear consciousness(indicates intact cognitive function sufficient to complete all required rating scales (23). Exclusion criteria: unstable condition, central pain due to other diseases (e.g., brain tumor, traumatic brain injury, spinal cord injury), coexisting peripheral neuropathy, pregnancy or lactation, severe cognitive impairment or intracranial metallic foreign bodies. The study was approved by the ethics committee of Hubei Third People’s Hospital, and all participants provided written informed consent. Patients were randomized into an acupuncture group and a combined treatment group. Each group consists of 15 cases, and the specific experimental design is shown in **Figure 1**.

### Interventions

All patients received conventional basic medical care and comprehensive rehabilitation including proper positioning of the affected limb, physical modalities, and transfer training, once daily, five times per week, for 4 weeks.

Acupuncture group: On the basis of routine care, patients received acupuncture on Jianyu (LI15), Quchi (LI11), Hegu (LI4), Zusanli (ST36), Waiguan (SJ5), Huantiao (GB30), Fengshi (GB31), Yanglingquan (GB34), Houxi (SI3), Chengshan (BL57), plus Baihui (GV20) on the Governor Vessel. Standardized Procedure: Following routine skin disinfection, sterile disposable needles were inserted perpendicularly using the balanced reinforcing-reducing technique. Successful needle manipulation was defined by the attainment of Deqi, characterized by (1): the practitioner’s perception of tenseness and dragging around the needle, and (2) the patient’s report of local soreness, numbness, distension, or heaviness. Needles were retained for 20 minutes following Deqi attainment.

Combined group: In addition to acupuncture as above, multi-target rTMS was administered using a Magneuro60 magnetic stimulator (Nanjing Weisi Medical Equipment Co., Ltd.) connected to a circular coil(diameter: 140 mm). The device specifications included a peak magnetic field: 6T, an output frequency of 60Hz(tolerance: ± 20%), an output frequency of 60 Hz (tolerance: ± 20%).

Resting motor threshold was determined by electromyography of the abductor pollicis brevis muscle (APB) in unaffected limb. The recording electrode was placed at the center of the APB belly, the reference electrode at the radial styloid process of the ipsilateral wrist with an interelectrode distance of ≥2 cm, and the ground electrode at the middle segment of the ipsilateral forearm. Motor evoked potential (MEP) at rest required ≥5 responses with peak-to-peak amplitude >50 μV out of 10 stimuli. Targets were localized using the international 10–20 EEG system. Magnetic stimulation target 1: contralateral primary motor corte M1 of the painful side, localized at C3/C4 contralateral to the painful side. Magnetic stimulation target 2: left DLPFC, localized at the F3 site of the left hemisphere. Stimulation parameters: frequency 10 Hz, intensity 80% RMT, inter-train interval 8 seconds, 20 trains, 900 pulses per magnetic target, once daily, Monday to Friday, for 4 consecutive weeks. Subjects received sequential stimulation of the contralateral M1 and left DLPFC within a single session, with the order randomized via computer-generated sequences to minimize potential order effects.

### Outcome measures

The primary outcome was pain severity, measured on a 10-point VAS (24) where scores of 1–3, 4–6, 7–9, and 10 represented mild, moderate, severe, and extremely severe pain, respectively. Psychological status, assessed by the Hamilton Depression Rating Scale (25) (HAMD), 24-item, range 0–76; and Hamilton Anxiety Rating Scale (26) (HAMA), 14-item, range 0–56; and sleep quality, assessed by Pittsburgh Sleep Quality Index (27)(PSQI), with scores >7 indicating clinically significant disturbance. Secondary outcomes include functional capacity, assessed by Activities of Daily Living (25) (ADL) scale, categorized as complete dependence (≤20), moderate dependence (40–55), or mild dependence(≥60).

### Statistical analysis

Data were analyzed using SPSS 26.0 software. Baseline demographic and clinical characteristics were compared between groups using independent t-tests or Mann-Whitney U tests for continuous variables (i.e., age, CPSP chronicity, and clinical scores) and chi-square tests for categorical variables (i.e., sex, stroke type, and pain side). A linear mixed model (LMM) was employed to analyze the effects of group, time, and their interaction on all outcome measures (VAS, HAMA, HAMD, PSQI, ADL). The model included group (acupuncture, combined), time (pre-treatment, post-treatment), and the group-by-time interaction as fixed effects. Participants were included as a random intercept to account for within-subject correlation over time. The model was adjusted for the baseline covariates of age, sex, CPSP chronicity, stroke type, and pain side. To further adjust for potential baseline imbalances and individual variability, the baseline value of each outcome was also included as a covariate in the model. This approach provides unbiased estimates of the group-by-time interaction, which reflects the differential treatment effect. Restricted maximum likelihood (REML) estimation was used. All statistical tests were two-tailed, and the alpha level for statistical significance was set at P<0.05.

## Results

### Baseline differences

Shapiro-Wilk tests were conducted to assess normality separately for each group (acupuncture group and combined group) for each scale at baseline. The results indicated that HAMA, HAMD, and PSQI scores were normally distributed in both groups (all P > 0.05), whereas VAS and ADL scores deviated significantly from normality in one or both groups (P < 0.05). Accordingly, baseline comparisons between groups were performed using independent t-tests for HAMA, HAMD, and PSQI, and Mann-Whitney U tests for VAS and ADL. No significant baseline differences were observed between the two groups in any outcome measure (all P > 0.05). Baseline demographic and clinical characteristics were also well balanced (all P > 0.05; **Table 1**). **Table 2** presents the descriptive statistics (Mean ± SD) for all outcome measures at baseline and after 4 weeks of intervention.

**Table 1:** Baseline characteristics of the two groups.

Variable	Acupuncture group (n=15)	Combined group (n=15)	Test statistic	P-value
Sex (M/F)	12/3	10/5	χ² = 0.682	0.409
Age (years)	60.12 ± 10.34	58.23 ± 9.67	t = 0.521	0.607
CPSP chronicity (months)	2.45 ± 1.23	2.15 ± 1.06	t = 0.728	0.473
Stroke type (ischemic/hemorrhagic)	10/5	9/6	χ² = 0.144	0.705
Pain side (left/right)	9/6	6/9	χ² = 1.200	0.273

CPSP, central post-stroke pain.

**Table 2:** Outcome measures at baseline and after 4-week intervention for both groups.

Outcome measure	Acupuncture group (n=15)		Combined group (n=15)	
	Pre-treatment	Post-treatment	Pre-treatment	Post-treatment
VAS	5.13 ± 0.52	2.80 ± 1.26	5.47 ± 0.92	1.87 ± 1.13
HAMA	26.27 ± 1.44	14.27 ± 1.98	26.13 ± 6.16	11.73 ± 2.49
HAMD	23.40 ± 1.50	14.13 ± 1.46	22.27 ± 2.87	10.27 ± 2.89
PSQI	8.07 ± 1.03	3.53 ± 1.85	7.53 ± 1.30	2.33 ± 1.11
ADL	62.33 ± 7.99	70.67 ± 5.63	60.67 ± 7.29	66.67 ± 5.23

Data are presented as Mean ± Standard Deviation (SD). Abbreviations (in alphabetical order): ADL, Activities of Daily Living; HAMA, Hamilton Anxiety Rating Scale; HAMD, Hamilton Depression Rating Scale; PSQI, Pittsburgh Sleep Quality Index; VAS, Visual Analogue Scale.

### Pain, mood, and sleep outcomes

**Figure 2:**
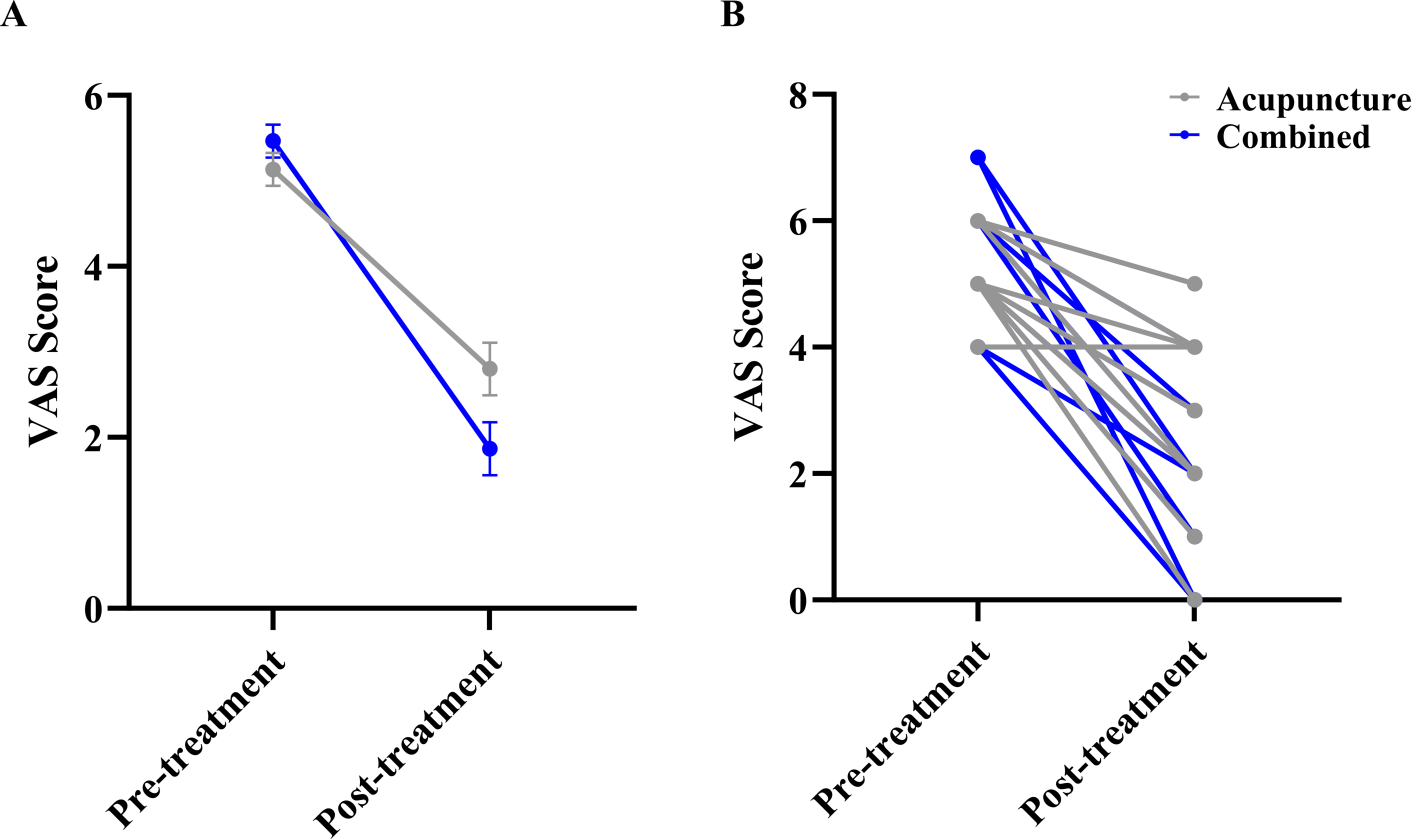
Comparison of visual analogue scale (VAS) scores between the two groups. **(A)** Estimated marginal means (EMM) with standard error (SE) bars from the linear mixed model, showing the main effect of Time and the Group × Time interaction. **(B)** Raw individual participant data trajectories. Each thin line connects the pre- and post-treatment scores of a single participant to visualize individual response variability.

The LMM results showed no significant main effect of Group (F (1, 280) = 1.297, P = 0.264). However, there was a significant main effect of Time (F (1, 280) =139.665, P < 0.001), indicating that VAS scores decreased significantly from pre- to post-treatment across both groups. Crucially, a significant Group × Time interaction was observed (F(1, 28.0) = 6.365, P = 0.018), demonstrating that the reduction in VAS scores was significantly greater in the combined group compared to the acupuncture group (**Figure 2A**). **Figure 2B** shows the individual participant data.

**Figure 3:**
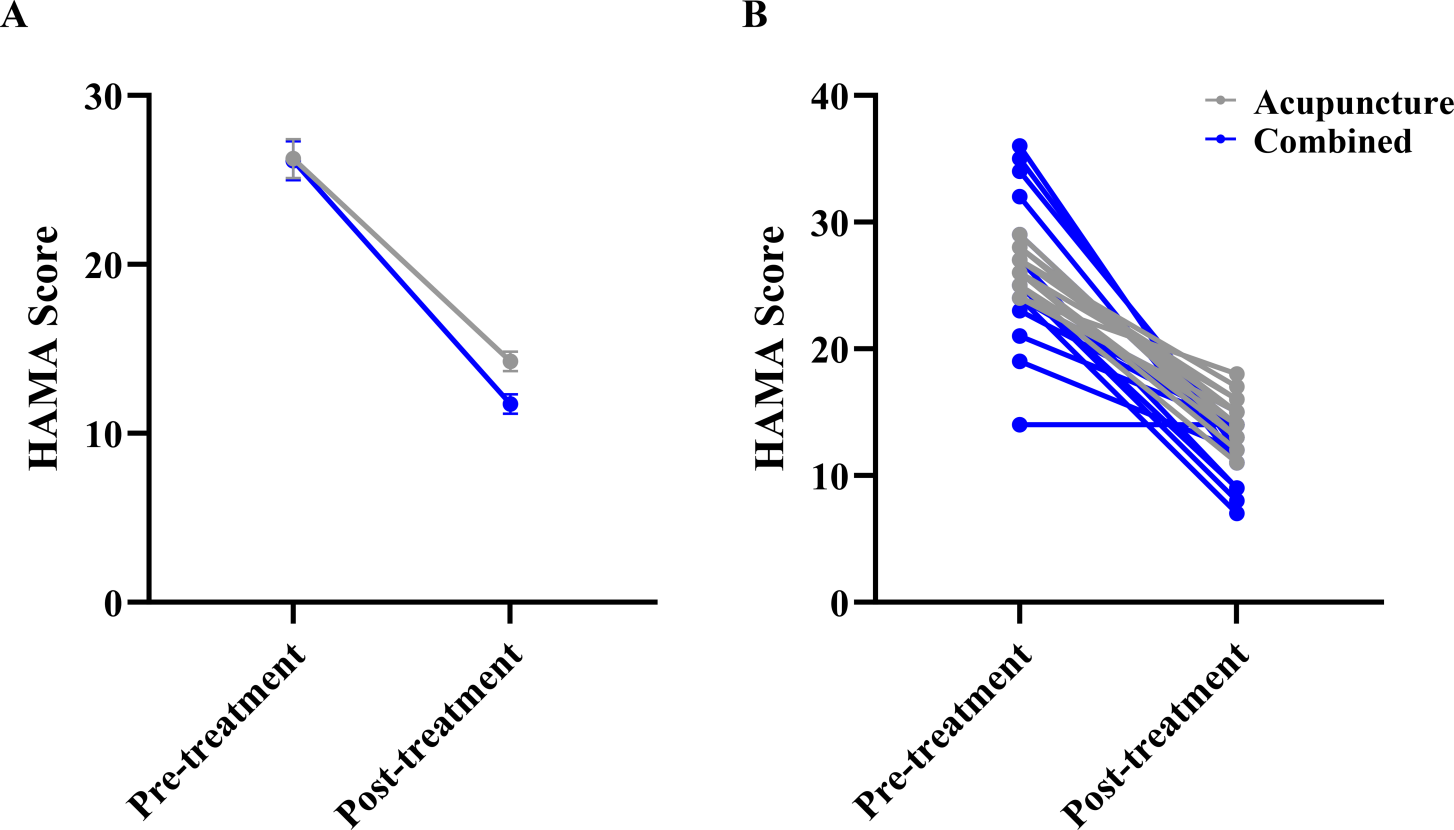
Comparison of Hamilton anxiety rating scale (HAMA) scores between the two groups. **(A)** Estimated marginal means (EMM) with standard error (SE) bars from the linear mixed model, showing the main effect of Time and the Group × Time interaction. **(B)** Raw individual participant data trajectories. Each thin line connects the pre- and post-treatment scores of a single participant to visualize individual response variability.

**Figure 4:**
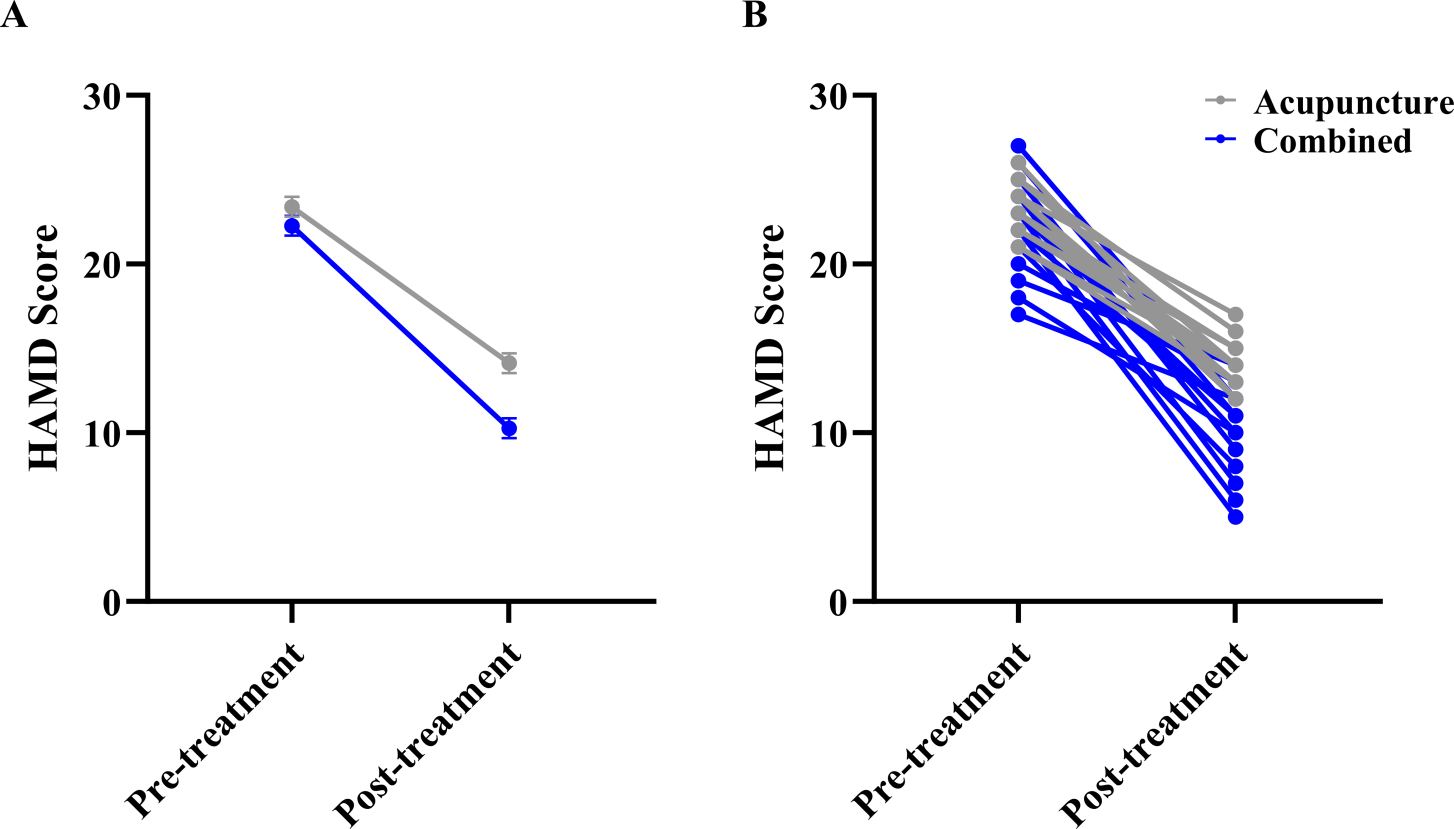
Comparison of Hamilton depression rating scale(HAMD) scores between the two groups. **(A)** Estimated marginal means (EMM) with standard error (SE) bars from the linear mixed model, showing the main effect of Time and the Group × Time interaction. **(B)** Raw individual participant data trajectories. Each thin line connects the pre- and post-treatment scores of a single participant to visualize individual response variability.

Regarding mood outcomes, LMM analysis for HAMA scores showed no significant main effect of Group (F(1,28.1)=2.426, P = 0.131) or Group × Time interaction (F(1,28.0)=1.532, P = 0.226), but a significant main effect of Time (F(1,28.0)=185.362, P<0.001), suggesting comparable improvements in anxiety between the two groups (**Figures 3A, B**). The LMM showed a significant main effect of Group for HAMD (F_(1, 28.0)_ = 20.550, P <0.001). A significant main effect of Time (F_(1,28.0)_=290.509, P<0.001), and a significant Group × Time interaction (F_(1,28.0)_=4.799, P = 0.037), indicating that the combined group experienced significantly greater reductions in depression scores than the acupuncture group (**Figures 4A, B**).

**Figure 5:**
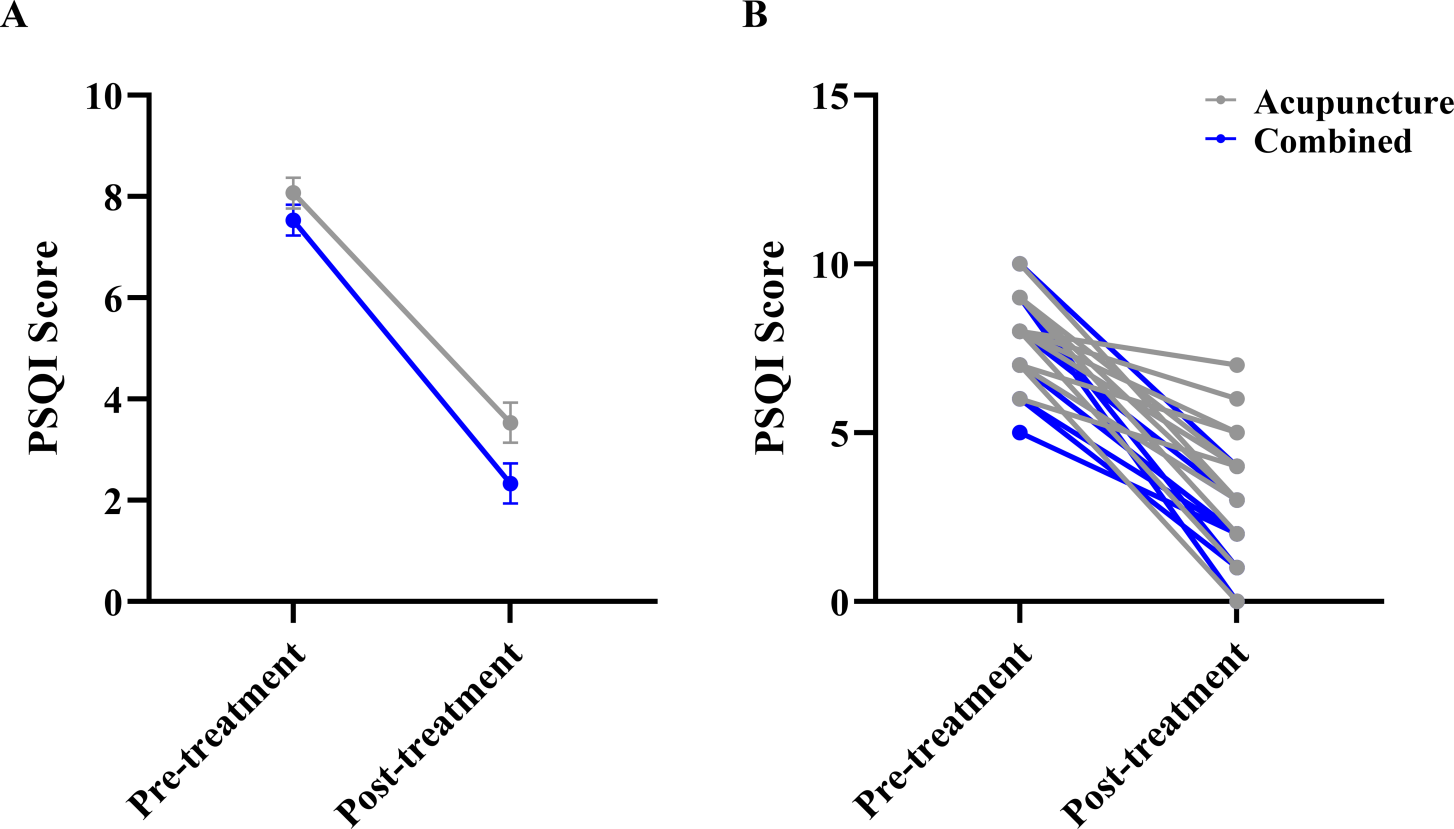
Comparison of Pittsburgh sleep quality index (PSQI) scores between the two groups. **(A)** Estimated marginal means (EMM) with standard error (SE) bars from the linear mixed model, showing the main effect of Time and the Group × Time interaction. **(B)** Raw individual participant data trajectories. Each thin line connects the pre- and post-treatment scores of a single participant to visualize individual response variability.

For sleep quality, the analysis of PSQI scores revealed no main effect of Group (F_(1, 28.0)_ = 5.878, P = 0.022). A significant main effect of Time (F_(1, 28.0)_ = 198.684, P < 0.001). However, the Group × Time interaction was not statistically significant (F_(1, 28.0)_ = 0.932, P = 0.343), indicating that the improvement in sleep quality did not differ significantly between groups (**Figures 5A, B**).

### Functional status

**Figure 6:**
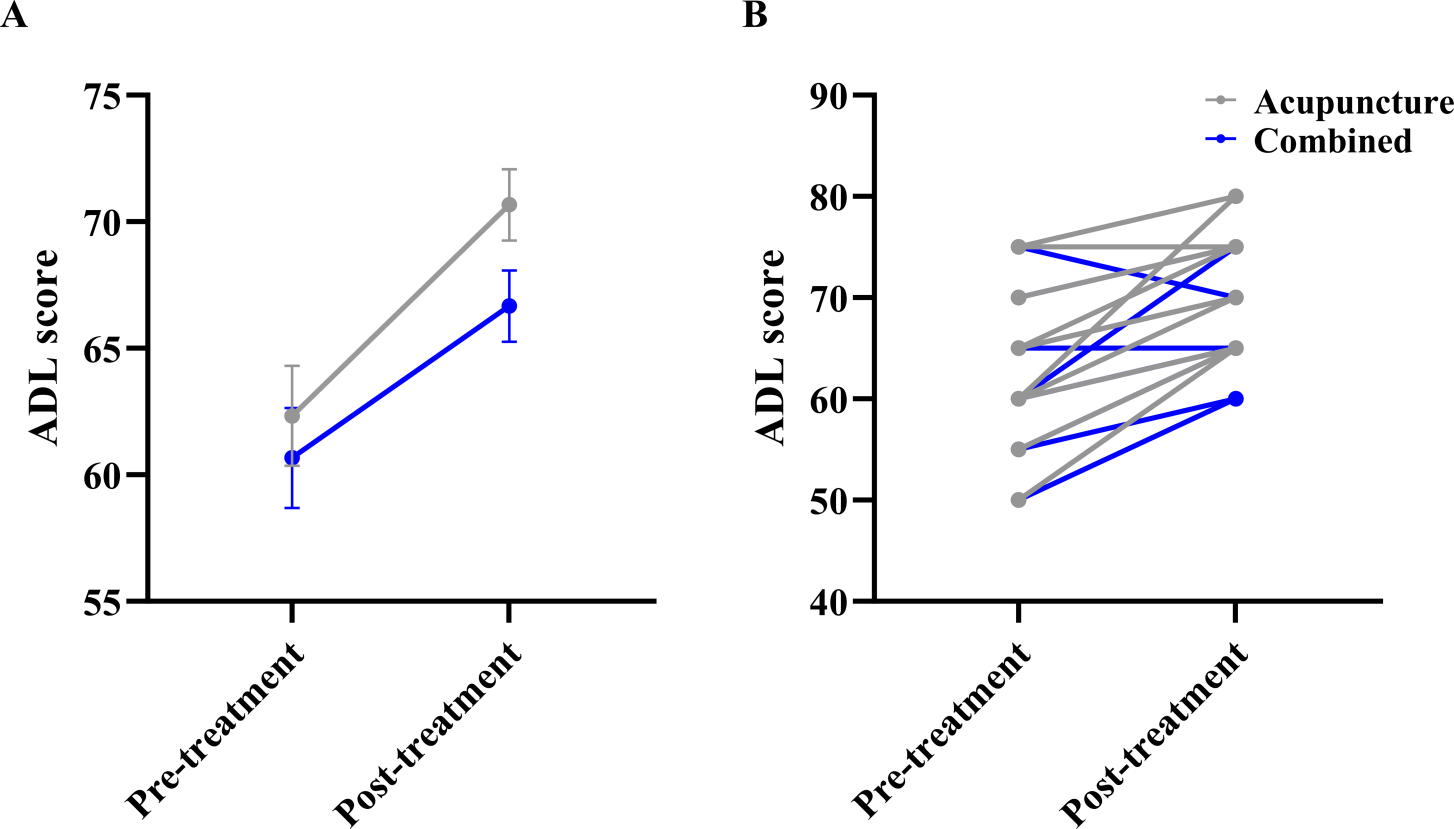
Comparison of activities of daily living (ADL) scores between the two groups. **(A)** Estimated marginal means (EMM) with standard error (SE) bars from the linear mixed model, showing the main effect of Time and the Group × Time interaction. **(B)** Raw individual participant data trajectories. Each thin line connects the pre- and post-treatment scores of a single participant to visualize individual response variability.

For activities of daily living, the LMM showed no main effect of Group (F_(1, 28.0)_ = 1.593, P = 0.217). A significant main effect of Time (F_(1, 28.0)_ = 62.226, P < 0.001) indicated that ADL scores improved significantly in both groups after 4 weeks. However, the Group × Time interaction was not statistically significant (F_(1, 28.0)_ = 1.649, P = 0.210), suggesting that the addition of rTMS did not lead to significantly greater functional improvements compared to acupuncture alone over the study period (**Figures 6A, B**).

## Discussion

This study aimed to investigate the clinical efficacy of multi-target HF-rTMS combined with acupuncture. The results demonstrated that, following the 4-week intervention, both groups exhibited significant improvements in clinical outcomes compared to baseline; notably, the combination group demonstrated significantly greater improvements in pain (VAS) and depression (HAMD) compared to the acupuncture-only group, whereas no significant between-group differences were observed in anxiety (HAMA), sleep quality (PSQI), or activities of daily living (ADL). These findings suggest that HF-rTMS targeting both the contralateral M1 and the left DLPFC, when integrated with acupuncture, confers synergistic advantages in pain relief and improvement in depressive symptoms.

This synergistic effect may arise from the complementary mechanisms of integrated traditional Chinese and Western medicine neuroregulation. First, HF-rTMS over the motor cortex may significantly reduces perceived intensity of neuropathic pain,

fibromyalgia and other chronic pain states (6). The underlying mechanisms may involve modulation of nociceptive transmission in the thalamus, as well as indirect effects on pain-related brain regions. DLPFC activity is inversely correlated with the unpleasant effect of pain, augmenting DLPFC function can indirectly inhibit nociception; together these interventions produce an analgesic synergy (19, 28, 29). Second, acupuncture-induced suppression of neuroinflammation (9, 29) and enhancement of cerebral perfusion (30) likely create additive effects with the cortical neuromodulation induced by rTMS. The absence of significant between-group differences in ADL improvements may be attributed to the relatively brief intervention duration (4 weeks), as functional recovery typically requires more prolonged neuroplastic remodeling and motor rehabilitation.

Our findings align with prior evidence demonstrating that high-frequency rTMS over the M1 significantly alleviates neuropathic pain (31), with 10 Hz protocols inducing more robust long-term potentiation of descending inhibitory pathways compared to lower frequencies (32, 33). Additionally, the documented negative correlation between DLPFC activity and pain affective distress supports the rationale that enhancing DLPFC function indirectly suppresses nociceptive processing (6). Acupuncture has likewise demonstrated significant efficacy in ameliorating post-stroke pathological pain and hemiplegic shoulder pain (7, 34). The novelty of the present study lies in the integration of multi-target rTMS with acupuncture, thereby expanding the therapeutic repertoire for combined neuromodulation. Compared with previous uni-modal interventions (14), the combination group in this study exhibited greater magnitude of improvement in pain severity and emotional comorbidities, suggesting that multi-modal therapeutic strategies may overcome the efficacy ceiling inherent to single-modality approaches.

There are some limitations to this trial. The lack of a non-acupuncture control group prevents us from ruling out spontaneous recovery effects. Furthermore, the combination group received increased clinical contact, this “additional attention effect” constitutes a potential confounder that obscures whether outcomes resulted from specific therapeutic mechanisms or non-specific contact frequency. We also did not account for potential heterogeneity between C3 and C4 stimulation sites, potentially limiting target-specific generalizability. Finally, the small sample size and brief follow-up may have constrained statistical power, preventing detection of ADL differences and long-term efficacy confirmation. In the future, we will optimize the research design, add control groups, control confounding factors, increase the sample size and perform stratified analysis, prolong the observation period, and simultaneously incorporate multimodal neuroimaging and electrophysiological indicators, thereby elucidating the mechanism of action and providing theoretical support for clinical translation.

## Data Availability

The original contributions presented in the study are included in the article/supplementary material. Further inquiries can be directed to the corresponding author.
