# Correction: Effects of high-frequency repetitive transcranial magnetic stimulation combined with acupuncture on central pain and mood

**DOI:** 10.3389/fpsyt.2026.1875233

**Published:** 2026-05-26

**Authors:** Xiangyue Wu, Binqing Li, Ting Xiang, Xiaolu Fan

**Affiliations:** 1 Department of Rehabilitation Medicine, The Third People’s Hospital of Hubei Province, Wuhan, Hubei, China; 2 Department of Gastrointestinal Surgery, Hubei Provincial Third People’s Hospital (Zhongshan Hospital), Wuhan, China

**Keywords:** acupuncture, central post-stroke pain, emotion, neuropathic pain, repetitive transcranial magnetic stimulation

Affiliation ‘Department of Gastrointestinal Surgery, Hubei Provincial Third People's Hospital (Zhongshan Hospital), Wuhan, China’ was omitted for author Ting Xiang. This affiliation has now been added for author Ting Xiang.

Author Ting Xiang was erroneously assigned to affiliation ‘Department of Rehabilitation Medicine, The Third People’s Hospital of Hubei Province, Wuhan, Hubei, China’. This affiliation has now been removed for author Ting Xiang.

The original version of this article has been updated.

